# The Efficiency of Split Panel Designs in an Analysis of Variance Model

**DOI:** 10.1371/journal.pone.0154913

**Published:** 2016-05-10

**Authors:** Xin Liu, Wei-Guo Wang, Hai-Jun Liu

**Affiliations:** 1 School of Economics and Management, Liaoning Shihua University, Fushun, China; 2 School of Economics, Dongbei University of Finance and Economics, Dalian, China; 3 School of Marxism, Dalian Maritime University, Dalian, China; University of Rijeka, CROATIA

## Abstract

We consider split panel design efficiency in analysis of variance models, that is, the determination of the cross-sections series optimal proportion in all samples, to minimize parametric best linear unbiased estimators of linear combination variances. An orthogonal matrix is constructed to obtain manageable expression of variances. On this basis, we derive a theorem for analyzing split panel design efficiency irrespective of interest and budget parameters. Additionally, relative estimator efficiency based on the split panel to an estimator based on a pure panel or a pure cross-section is present. The analysis shows that the gains from split panel can be quite substantial. We further consider the efficiency of split panel design, given a budget, and transform it to a constrained nonlinear integer programming. Specifically, an efficient algorithm is designed to solve the constrained nonlinear integer programming. Moreover, we combine one at time designs and factorial designs to illustrate the algorithm’s efficiency with an empirical example concerning monthly consumer expenditure on food in 1985, in the Netherlands, and the efficient ranges of the algorithm parameters are given to ensure a good solution.

## Introduction

Split panel combines advantages of other sampling methods (including repeated, cross-sectional, and rotating sample) and provides us with rich, convenient, and practical information, being widely applied in many fields [1]. In experiments with economic principles survey, researchers typically consider statistical models that allow complex relationships. Due to the complex statistical model requirements, data should be collected to estimate the statistical model parameters. Being a new sample type that combines the advantages of the other three basic samples, split panel is used to provide rich data for complex statistical model. Since wide application of micro-economic data, panel conditioning, and panel nonresponse become more important in econometrics, as well documented in literature [1], split panel, as a combination of a panel and a repeated or rotating panel, uses changing samples to recruit from replacements for panel conditioning and panel nonresponse [2–3]. In most fields, such as finance, labor economics, and political economy, the collection of data is characterized by high costs. Split panel has the advantages of the flexibility of the cross-section change sizes and continually updating information [4–5]. Therefore, it is very important to use split panel and the optimal sample design to obtain as much information as possible from a given budget. In recent years, the theory of split panel sample has witnessed theoretical advances and applications across disciplines of pure and applied sciences, and it will be widely used in the future [6–7]. However, limited attention has been paid to the analysis of split panel design efficiency recently. In the early literature, the estimation of a time-dependent mean from several kinds of rotating samples, that is, the special form of split panel and the resulting variances have been examined by Patterson [8] and Eckler [9]. It has been documented that the optimal design of the sample depends on the parameter of interest (see [10], pp. 152). On this basis, Nijman et al. [11] determined the optimal split panel design, that is, how to choose the optimal proportion of a given budget that can be spent on the collection of a series of cross-sections to minimize split panel design efficiency. However, in sampling, we need to obtain the optimal proportion of a series of cross-sections in all samples, and it cannot be obtained accurately by the proportion of the budget that can be spent on the collection of a series of cross-sections [11]. Consequently, we cannot save sampling costs according to [11]. On the other hand, the split panel design optimization algorithm is not given in [11]. For researchers and practitioners to solve for the optimal split panel design, they need to select or design the appropriate optimization algorithm and to calculate the optimal proportion by the optimization theory. In sampling, this will decrease the efficiency of calculating the optimal proportion and reduce the accuracy of the solution. Hence, it is not attractive to design split panel in the research framework of [11].

In this paper, the goal is to minimize the efficiency of split panel design in the analysis-of-variance model, when one needs to determine the optimal proportion of a series of cross-sections in all samples, when the optimal proportion of a series of cross-sections can be applied directly in sampling. This is an extension of [11], and the main contributions can be summarized as follows. First, we show how to choose the proportion of a series of cross-sections, in all samples, to minimize the variances of estimators in the analysis-of-variance model, irrespective of the parameters of interest and budget. In particular, we present the relative efficiency of estimators, based on the split panel to an estimator based on a pure panel or a pure cross-section. Second, we transform the efficiency of split panel design under a budget constraint for the nonlinear integer optimization (difficult to solve by mature optimal algorithms). The simulated annealing algorithm has the advantages of guaranteeing global optimization, selecting the initial solution randomly, and being simple and practical [12]. However, when the simulated annealing algorithm is used to solve the constrained nonlinear integer optimization associated with the efficiency of split panel design under a budget constraint, it is difficult to combine parameters, such as the inner iteration number, the initial temperature, and the temperature decrease rate, in order to get the best performance of the algorithm. Hitherto, there is no theoretical method to solve this problem. Therefore, in this paper we design an efficient algorithm, based on the simulated annealing algorithm, to solve the constrained nonlinear integer optimization of the split panel design efficiency under budget constraint. In the context of numerical modeling, sensitivity analysis studies how different values of an independent variable impact a particular dependent variable, under a given set of assumptions. It has been widely applied to many fields such economics, engineering, ecology, etc. The modelers can determine, by sensitivity analysis, whether the parameters of the model or algorithm give reliable predictions. Hence, third, we introduce sensitivity analysis to appraise the parameters of the proposed algorithm. The one-at-a-time design (OATD) method is one of the most common approaches for the effect on the output [13–16] and it is frequently used as the modeler immediately knows which input factor is responsible for the failure, in case of model failure [17]. Yet, the OATD method cannot be used if two factors are interdependent, because it only studies the effect of one variable at a time. The factorial design (FD) method, which is used to study the effects several factors have on a response, and the interactions between the factors for varying levels of all factors at the same time, is different from the OATD method. As such, the OATD and FD methods are chosen to analyze the effect of parameters and compensate the deficiency of a single method [16]. On the other hand, the simulated annealing algorithm has no special requirement and its performance cannot be changed with different examples [12]. Hence, with an empirical example concerning monthly consumer expenditure on food in 1985, in the Netherlands, we combine the OATD and FD methods to analyze the algorithm designed in this paper. The result are the efficient ranges of the algorithm parameters are a good solution (i.e., the accurate optimal proportion). Therefore, the research results in this paper would be useful to both researchers and practitioners in sampling.

This paper is organized as follows: section 2, based on the analysis-of-variance model, transforms the efficiency of split panel design into a nonlinear optimization; in section 3, the efficiency of split panel design, irrespective of interest and budget, is discussed; in section 4, we consider the efficiency of split panel design given a budget constraint and design an efficient algorithm based on simulated annealing to solve the resulting constrained nonlinear integer optimization; section 5 combines the OATD and FD methods to illustrate the algorithm’s efficiency, with an empirical example of food monthly consumer expenditure in 1985, in the Netherlands, and the efficient ranges of the algorithm parameters are given to ensure a good solution; and section 6 concludes the paper.

## Materials and Methods

### Theoretical results of parameter estimators variances

In this paper, we consider the split panel design efficiency by minimizing the best linear estimator variance of the linear combinations $\sum_{t=1}^{T}\phi_{t}{\overset{⌢}{\beta }}_{t}$ of the period means ${\overset{⌢}{\beta }}_{t}$ in the analysis of the variance model:

$$y_{it}=\beta_{t}+\alpha_{i}+\varepsilon_{it},$$ (1)

where *i* = 1,…,*N*, *t* = 1,…,*T* and *ϕ*′ = (*ϕ*_1_,*ϕ*_2_…,*ϕ*_*T*_), the *α*_*i*_ and *ε*_*it*_ are independent and identically distributed (i.i.d.) random variables with mean 0 and variances $\sigma_{\alpha }^{2}$ and $\sigma_{\varepsilon }^{2}$, respectively, which are mutually independent. Throughout this paper we assumed that the parameters $\sigma_{\alpha }^{2}$ and $\sigma_{\varepsilon }^{2}$ are a priori known, for simplicity. If these parameters are unknown, the consistent estimators can be used in their place and the same results hold true asymptotically [18]. Important special cases are the determination of the optimal design if the parameter of interest is the period mean *β*_*t*_, if the parameter of interest is the change in two subsequent period means *β*_*t*_−*β*_*t−*1_, or if the parameter of interest is the overall average of the period means $\sum_{t=1}^{T}\beta_{t}$.

We denote the sample size in each wave by *N* and the proportion of cross-sections in all samples by *λN*, while the remaining (1−*λ*)*N* individuals will be re-interviewed every period. In order to determine the optimal value of *λ* (i.e., the proportion of cross-sections in all samples) we first derive the efficient estimator and its variance. It is well known that the estimator of *β*′ = (*β*_1_,*β*_2_,…,*β*_*T*_) in Eq (1), using only the information on individuals which are re-interviewed every period, is the best linear unbiased estimator and regarded as ${\overset{⌢}{\beta }}_{p}$ [11]. Analogously, the estimator based on the cross-section information only is also the best linear unbiased estimator and regarded as ${\overset{⌢}{\beta }}_{cs}$ [11], and that

$$\text{var}({\overset{⌢}{\beta }}_{t})=\frac{1}{J^{2}}\text{var}(\sum_{i=1}^{J}\alpha_{i}+\sum_{i=1}^{J}\varepsilon_{it})=\frac{\sigma_{\varepsilon }^{2}+\sigma_{\alpha }^{2}}{J},$$ (2)

$$\text{cov}({\overset{⌢}{\beta }}_{t},{\overset{⌢}{\beta }}_{s})=\frac{1}{J^{2}}\text{cov}(\sum_{i=1}^{J}\alpha_{i}+\sum_{i=1}^{J}\varepsilon_{it},\sum_{i=1}^{J}\alpha_{i}+\sum_{i=1}^{J}\varepsilon_{is})=\frac{\sigma_{\alpha }^{2}}{J},(s\neq t)$$ (3)

where *J* denotes the number of observed individuals. Therefore

$$\text{var}({\overset{⌢}{\beta }}_{p})=\frac{1}{(1-\lambda )N}(\sigma_{\varepsilon }^{2}I_{T}+\sigma_{\alpha }^{2}l_{T}l_{T}{}^{\prime })=\frac{1}{(1-\lambda )N}V_{p},$$ (4)

$$\text{var}({\overset{⌢}{\beta }}_{cs})=\frac{1}{\lambda N}(\sigma_{\varepsilon }^{2}+\sigma_{\alpha }^{2})I_{T}=\frac{1}{\lambda N}V_{cs},$$ (5)

where *l*_*T*_ = (1,…,1)_*T*×1_′ and $V_{p}=\sigma_{\varepsilon }^{2}I_{T}+\sigma_{\alpha }^{2}l_{T}l_{T}{}^{\prime }$, $V_{cs}=\left(\sigma_{\varepsilon }^{2}+\sigma_{\alpha }^{2}\right)I_{T}$.

Since ${\overset{⌢}{\beta }}_{p}$ and ${\overset{⌢}{\beta }}_{cs}$ are independent, based on the relative theory of two sample estimation [10, 11], the best linear unbiased estimator which uses all the samples is given by

$$\overset{⌢}{\beta }={\left[(1-\lambda )NV_{p}^{-1}+\lambda NV_{cs}^{-1}\right]}^{-1}\left[(1-\lambda )NV_{p}^{-1}{\overset{⌢}{\beta }}_{p}+\lambda NV_{cs}^{-1}{\overset{⌢}{\beta }}_{cs}\right].$$ (6)

For

$$\text{var}\left[(1-\lambda )NV_{p}^{-1}{\overset{⌢}{\beta }}_{p}+\lambda NV_{cs}^{-1}{\overset{⌢}{\beta }}_{cs}\right]=(1-\lambda )NV_{p}^{-1}+\lambda NV_{cs}^{-1},$$ (7)

it is easily verified that

$$\text{var}\left(\phi^{\prime }\overset{⌢}{\beta }\right)=N^{-1}\phi^{\prime }{\left\{\lambda V_{cs}^{-1}+(1-\lambda )V_{p}^{-1}\right\}}^{-1}\phi .$$ (8)

Consequently, the efficiency of split panel design could be transformed into the following nonlinear optimization by minimizing the variance of the best unbiased estimator of $\phi^{\prime }\overset{⌢}{\beta }$

$$\underset{N,\lambda }{\text{min}}N^{-1}\phi^{\prime }{\left\{\lambda V_{cs}^{-1}+(1-\lambda )V_{p}^{-1}\right\}}^{-1}\phi .$$ (9)

In order to obtain the optimal solution for *λ* from Eq (9), the main steps are discussed in the derivation of the manageable expression for the variance of the best unbiased estimator $\phi^{\prime }\overset{⌢}{\beta }$ in Eq (1). First, $V_{p}^{-1}$ and $V_{cs}^{-1}$ can be written as

$$V_{p}^{-1}=\frac{1}{\left(\sigma_{\varepsilon }^{2}+\sigma_{\alpha }^{2}\right)}V_{p_{0}}^{-1}$$ (10)

and

$$V_{cs}^{-1}=\frac{1}{\left(\sigma_{\varepsilon }^{2}+\sigma_{\alpha }^{2}\right)}I_{T},$$ (11)

where

$$V_{p_{0}}^{-1}=\left[\begin{array}{cccc} \frac{(2-T)\rho -1}{(\rho -1)\left[1+(T-1)\rho \right]} & \frac{\rho }{(\rho -1)\left[1+(T-1)\rho \right]} & \ldots & \frac{\rho }{(\rho -1)\left[1+(T-1)\rho \right]}\\ \frac{\rho }{(\rho -1)\left[1+(T-1)\rho \right]} & \frac{1+(T-2)\rho }{(1-\rho )\left[1+(T-1)\rho \right]} & \ldots & \frac{\rho }{(\rho -1)\left[1+(T-1)\rho \right]}\\ \vdots & \vdots & \ddots & \vdots \\ \frac{\rho }{(\rho -1)\left[1+(T-1)\rho \right]} & \frac{\rho }{(\rho -1)\left[1+(T-1)\rho \right]} & \ldots & \frac{1+(T-2)\rho }{(1-\rho )\left[1+(T-1)\rho \right]} \end{array}\right]$$ (12)

and

$$\rho =\frac{\sigma_{\alpha }^{2}}{\left(\sigma_{\varepsilon }^{2}+\sigma_{\alpha }^{2}\right)}.$$ (13)

Since $V_{cs}^{-1}$ is a constant and multiple of the identity matrix, and $V_{p}^{-1}$ is symmetric, there exists an orthogonal matrix *Q* such that $Q^{T}V_{p_{0}}^{-1}Q=D$ and $Q^{T}V_{cs}^{-1}Q=\frac{1}{\left(\sigma_{\varepsilon }^{2}+\sigma_{\alpha }^{2}\right)}I_{T}$, where *D* is a diagonal matrix and written as

$$D=\left[\begin{array}{cccc} \frac{1}{1+(T-1)\rho } & 0 & \cdots & 0\\ 0 & \frac{1}{1-\rho } & \cdots & 0\\ \vdots & \vdots & \ddots & 0\\ 0 & 0 & 0 & \frac{1}{1-\rho } \end{array}\right],$$ (14)

and the orthogonal matrix *Q* can be written as

$$Q=\left[\begin{array}{cccc} \frac{-1}{\sqrt{T}} & \frac{-1}{\sqrt{T}} & \cdots & \frac{-1}{\sqrt{T}}\\ \frac{-1}{\sqrt{T}} & \frac{1+\sqrt{T}-T}{T-\sqrt{T}} & \cdots & \frac{1}{T-\sqrt{T}}\\ \vdots & \vdots & \ddots & \vdots \\ \frac{-1}{\sqrt{T}} & \frac{1}{T-\sqrt{T}} & \cdots & \frac{1+\sqrt{T}-T}{T-\sqrt{T}} \end{array}\right].$$ (15)

The proof of constructing the orthogonal matrix *Q* is presented in the Appendix A1.

As such, the variance of the best unbiased estimator of $\phi^{\prime }\overset{⌢}{\beta }$ using all the samples is written as

$$\text{var}\left(\phi^{\prime }\overset{⌢}{\beta }\right)==N^{-1}(\sigma_{\alpha }^{2}+\sigma_{\varepsilon }^{2})\phi^{\prime }Q^{\prime }{\left\{\lambda I+(1-\lambda )D\right\}}^{-1}Q\phi ,$$ (16)

where

$${\left\{\lambda I+(1-\lambda )D\right\}}^{-1}=\left[\begin{array}{cccc} \frac{1+(T-1)\rho }{1+(T-1)\rho \lambda } & 0 & \cdots & 0\\ 0 & \frac{1-\rho }{1-\lambda \rho } & \cdots & 0\\ \vdots & \vdots & \ddots & 0\\ 0 & 0 & 0 & \frac{1-\rho }{1-\lambda \rho } \end{array}\right].$$ (17)

We denote *ϕ*′*Q* = (*δ*_1_,…,*δ*_*T*_) = *δ*′ to obtain the simple expression of Eq (16), and rewrite it as

$$\text{var}\left(\phi^{\prime }\overset{⌢}{\beta }\right)=N^{-1}(\sigma_{\alpha }^{2}+\sigma_{\varepsilon }^{2})(\frac{1+(T-1)\rho }{1+(T-1)\lambda \rho }\delta_{1}^{2}+\sum_{t=2}^{T}(\frac{1-\rho }{1-\lambda \rho })\delta_{t}^{2}).$$ (18)

Consequently, the nonlinear optimization by minimizing the variance of the best unbiased estimator of $\phi^{\prime }\overset{⌢}{\beta }$ can be rewritten as

$$\underset{N,\lambda }{\text{min}}N^{-1}(\sigma_{\alpha }^{2}+\sigma_{\varepsilon }^{2})(\frac{1+(T-1)\rho }{1+(T-1)\lambda \rho }\delta_{1}^{2}+\sum_{t=2}^{T}(\frac{1-\rho }{1-\lambda \rho })\delta_{t}^{2}).$$ (19)

### Split panel design efficiency irrespective of the parameters of interest and budget

By considering the linear combinations of vector *β*, we can then easily adapt the results to an individual element, difference of elements, or overall average. As such, in this section, we will derive a theorem for the split panel design efficiency, irrespective of the parameters of interest and budget, using Eq (19).

**Theorem 1** Pure panel (*λ* = 0) will minimize the variance of the best unbiased estimator of $\phi^{\prime }\overset{⌢}{\beta }$, irrespective of the choice of *ϕ*,

$$\text{if }\frac{{\left(\sum_{t=1}^{T}\phi_{t}\right)}^{2}}{\sum_{t=1}^{T}\phi_{t}^{2}}<\frac{1-\rho }{1+T\rho -2\rho };$$ (20)

pure series of cross-sections (*λ* = 1) will minimize the variance of the best unbiased estimator of $\phi^{\prime }\overset{⌢}{\beta }$, irrespective of the choice of *ϕ*,

$$\text{if }\frac{{\left(\sum_{t=1}^{T}\phi_{t}\right)}^{2}}{\sum_{t=1}^{T}\phi_{t}^{2}}>\text{max}\left\{\frac{1-\rho }{1+T\rho -2\rho },1+T\rho -\rho \right\};$$ (21)

split panel (*λ* = k_*r*_) will minimize the variance of the best unbiased estimator of $\phi^{\prime }\overset{⌢}{\beta }$, irrespective of the choice of *ϕ*,

$$\text{if}\frac{1-\rho }{1+T\rho -2\rho }<\frac{{\left(\sum_{t=1}^{T}\phi_{t}\right)}^{2}}{\sum_{t=1}^{T}\phi_{t}^{2}}<\text{min}\left\{(1-\rho )(T-1),1+T\rho -\rho \right\};$$ (22)

split panel (*λ* = k_*l*_) will minimize the variance of the best unbiased estimator of $\phi^{\prime }\overset{⌢}{\beta }$. irrespective of the choice of *ϕ*,

$$\text{if }(1-\rho )(T-1)<\frac{{\left(\sum_{t=1}^{T}\phi_{t}\right)}^{2}}{\sum_{t=1}^{T}\phi_{t}^{2}}<1+T\rho -\rho ;$$ (23)

split panel (*λ* = *λ*_0_) will minimize the variance of the best unbiased estimator of $\phi^{\prime }\overset{⌢}{\beta }$, irrespective of the choice of *ϕ*,

$$\text{if }\frac{{\left(\sum_{t=1}^{T}\phi_{t}\right)}^{2}}{\sum_{t=1}^{T}\phi_{t}^{2}}=(1-\rho )(T-1),$$ (24)

where k_*l*_ is the left root of k(*λ*); k_*r*_ is the right root of k(*λ*),

$$k(\lambda )=(1-T)\rho A{\left(1-\lambda \rho \right)}^{2}+B\rho {\left(1+(T-1)\lambda \rho \right)}^{2}.$$ (25)

If

$$M(-\frac{c}{b})<M(1),\lambda_{0}=-\frac{c}{b},$$ (26)

if

$$M(-\frac{c}{b})>M(1),\lambda_{0}=1,$$ (27)

And

$$b=2\rho^{2}(T-1)\left[(1-\rho )\sum_{t=1}^{T}\varphi_{t}^{2}+\rho {\left(\sum_{t=1}^{T}\varphi_{t}\right)}^{2}\right],$$ (28)

$$c=(1-\rho )\rho \sum_{t=1}^{T}\varphi_{t}^{2}+\rho (2\rho -1-\rho T){\left(\sum_{t=1}^{T}\varphi_{t}\right)}^{2}.$$ (29)

$$M(\lambda )=\frac{(1-\lambda )\rho }{\left(1+(T-1)\lambda \rho )(1-\lambda \rho \right)}{\left(\sum_{t=1}^{T}\varphi_{t}\right)}^{2}+\frac{1-\rho }{1-\lambda \rho }\sum_{t=1}^{T}\varphi_{t}^{2}.$$ (30)

The proof of theorem 1 is presented in Appendix 1B.

From Theorem 1, it can be easily checked that ${\overset{⌢}{\beta }}_{t}-{\overset{⌢}{\beta }}_{t-1}$ has the smallest variance if a pure panel (*λ* = 0) is used. Likewise, a pure series of cross-sections (*λ* = 1) will be optimal if the overall average of period means is to be estimated.

In order to illustrate that the split panel design will be preferable to pure panel or pure series of cross-sections design in most cases, and how much efficiency will be lost if a suboptimal choice is made when the period mean *β*_*t*_ is the parameter of interest, we present in Table 1 the relative efficiency of the estimator based on the split panel to an estimator based on a pure series of cross-sections or pure panel (pure series of cross-sections and pure panel yield equally efficient estimators in this case). Similar to [18], we assume the observation period *T* = 3,6,12,20, the proportion of the component of variance *ρ* = 0.3,0.6,0.9, and the proportions of a series of cross-sections in split panel *λ* = 1/2,1/3,1/4,1/8,1/12.

**Table 1:** The relative efficiency compared to pure cross-sections (or pure panel) for the estimator ${\overset{⌢}{\beta }}_{t}$.

	*ρ*	*T* = 3	*T* = 6	*T* = 12	*T* = 20
***λ* = 1/2**	***0*.*3***	***0*.*9593***	***0*.*9244***	***0*.*8901***	***0*.*8694***
	***0*.*6***	***0*.*8393***	***0*.*7429***	***0*.*6711***	***0*.*6354***
	***0*.*9***	***0*.*6124***	***0*.*4336***	***0*.*3193***	***0*.*2675***
***λ* = 1/3**	***0*.*3***	***0*.*9630***	***0*.*9259***	***0*.*8836***	***0*.*8544***
	***0*.*6***	***0*.*8571***	***0*.*7500***	***0*.*6563***	***0*.*6042***
	***0*.*9***	***0*.*6786***	***0*.*4857***	***0*.*3422***	***0*.*2708***
***λ* = 1/4**	***0*.*3***	***0*.*9683***	***0*.*9337***	***0*.*8900***	***0*.*8571***
	***0*.*6***	***0*.*8778***	***0*.*7731***	***0*.*6704***	***0*.*6081***
	***0*.*9***	***0*.*7297***	***0*.*5389***	***0*.*3797***	***0*.*2941***
***λ* = 1/8**	***0*.*3***	***0*.*9810***	***0*.*9569***	***0*.*9204***	***0*.*8865***
	***0*.*6***	***0*.*9260***	***0*.*8452***	***0*.*7434***	***0*.*6665***
	***0*.*9***	***0*.*8370***	***0*.*6806***	***0*.*5092***	***0*.*3955***
***λ* = 1/12**	***0*.*3***	***0*.*9866***	***0*.*9687***	***0*.*9392***	***0*.*9092***
	***0*.*6***	***0*.*9474***	***0*.*8842***	***0*.*7946***	***0*.*7179***
	***0*.*9***	***0*.*8837***	***0*.*7568***	***0*.*5968***	***0*.*4759***

### Split panel design efficiency with budget constraint and main algorithm

As opposed to the previous section, where we have analyzed split panel design efficiency, irrespective of the parameters of interest and budget, in this section, split panel design efficiency with a given budget is considered. Let *p*_1_ denote the average cost of observing every individual in cross-sections and *p*_2_ the average cost of observing every individual in panels. The cost of a cross-sectional survey is 30% to 70% higher than an additional wave of the panel study of income dynamics, as shown by Duncan et al. [19]. Therefore, we obtain $0.6<\frac{{}_{p_{2}}}{p_{1}}<0.8$. If there is a budget,*C*, for all the periods, we can obtain the constrained nonlinear integer optimization (P1), as follows

$$\text{min}z(\lambda ,N)=N^{-1}\phi^{\prime }{\left\{\lambda V_{cs}^{-1}+(1-\lambda )V_{p}^{-1}\right\}}^{-1}\phi ,$$ (31)

$$s.t.\;\lambda Np_{1}+(1-\lambda )Np_{2}\leq C^{*},$$ (32)

0≤λ≤1, (33)

$$\lambda N,N\in N^{*},$$ (34)

where $C^{*}=\frac{C}{T}$.

Applying Eq (19) and *λN* = *x*, *N* = *y*, we obtain the constrained nonlinear integer optimization (P2):

$$\underset{x,y}{\text{min}}z(x,y)=\sigma^{2}\cdot (\frac{1+(T-1)\rho }{y+(T-1)x\rho }\delta_{1}^{2}+\sum_{t=2}^{T}(\frac{1-\rho }{y-x\rho })\delta_{t}^{2}),$$ (35)

$$\text{s.t}.\;x(p_{1}-p_{2})+yp_{2}\leq C^{*},$$ (36)

0≤x≤y, (37)

$$x,y\in N^{*},$$ (38)

where $C^{*}=\frac{C}{T}$.

Eq (35) is the objective function that minimizes the variance of the best linear unbiased estimator of linear combinations of the period means, while Eq (36) satisfies the constraint of a given budget.

#### Algorithm design

In section 4, we transformed the efficiency of split panel design into the constrained nonlinear integer optimization (P2), which is, nonetheless, difficult to solve with the current mature optimal algorithms. The simulated annealing algorithm has the advantages of guaranteeing global optimization, selecting the initial solution randomly, while being simple and practical. However, when it is used to solve the constrained nonlinear integer optimization associated with the efficiency of split panel design, given a budget, it is difficult to combine the parameters, such as the inner iteration number, the initial temperature, and the temperature decrease rate, in order to get the best performance of the algorithm. Consequently, in this paper, we design an efficient algorithm to solve the constrained nonlinear integer optimization associated with split panel design efficiency of given a budget, based on the simulated annealing algorithm.

The steps of the simulated annealing algorithm designed to solve (P2) are given as follows:

1. Choose the initial integer solution *x*_0_ ∈ *D* and the initial temperature value *T*_0_>0, where *D* is a feasible region formed by Eqs (4) and (5); calculate *f*(*x*_0_) and let
  $$X_{0}=x_{0},X_{\text{min}}=X^{0},f_{\text{min}}=f(x^{0}),K=0,$$ (39)
2. Randomly generate the integer vector
  $$Z^{K}=(z_{1}^{K},\cdots ,z_{n}^{K}),$$ (40)
  where
  $$z_{i}^{K}=\langle sign(U_{i})\cdot T_{K}\cdot (\frac{1}{{\left|U_{i}\right|}^{m}}-1)\rangle ,i=1,2,\cdots ,n,$$ (41)
  and $z_{i}^{K}$ is the ith component of the random vector *Z*^*K*^; *U*_1_,*U*_2_,…,*U*_*n*_ is a group of random variables distributed uniformly over [−1,1], which are independent each other; *sign*(⋅) is the sign function; and 〈⋅〉 is the symbol of rounding numbers.
3. Use the current iteration point *x*^*K*^ and the random vector *Z*^*K*^ to generate a new iteration point *Y*^*K*^ that satisfies *Y*^*K*^ = *X*^*K*^+*Z*^*K*^. If *Y*^*K*^∈*D*, the next step is carried on, and if *Y*^*K*^∉*D*, *Y*^*K*^ is calculated by
  $$Y^{K}=X^{K}+\langle {\left(\frac{-1}{2}\right)}^{l}\cdot Z^{K}\rangle$$ (42)
  until *Y*^*K*^∈*D* and to the next step, where *l* = 1,2,…*N*_1_. If *Y*^*K*^∉*D* in the *N*_1_ steps, let *Y*^*K*^ = *X*^*K*^ and move to the next step.
4. Generate a random number *η* distributed uniformly over [0,1] and calculate
  $$P_{a}(Y^{K}|X^{K},T_{K})=\text{min}\left\{1,\text{exp}(\frac{f(X^{K})-f(Y^{K})}{\beta T_{K}})\right\}$$ (43)
  using the current iteration point *X*^*K*^ and a new iteration point, *Y*^*K*^.
5. If
  $$\eta \leq P_{a}(Y^{K}|X^{K},T_{K}),$$ (44)
  let
  $$X^{K+1}=Y^{K},f(X^{K+1})=f(Y^{K})$$ (45)
  or let
  $$X^{K+1}=X^{K},f(X^{K+1})=f(X^{K}).$$ (46)
6. If
  $$f(X^{K+1})<f_{\text{min}},$$ (47)
  let
  $$X_{\text{min}}=X^{K+1},f_{\text{min}}=f(X^{K+1}).$$ (48)
7. If the stopping criterion satisfies
  $$\left|f(X^{K+1})-f(X^{K})\right|<\varepsilon ,$$ (49)
  stop calculating and regard *X*_min_ and *f*_min_ as the approximate global optimal solution and the corresponding optimal value, respectively. If not, move to the next step.
8. Generate a new temperature *T*_*K*+1_ by using the given renewed function of temperature as follows:
  $$T_{K+1}=\frac{T_{0}}{{\left(K+1\right)}^{m}},K=0,1,2\cdots$$ (50)
  and let *K* = *K+*1 and shift to the second step.

The detailed design process of simulated annealing algorithm is presented in Appendix 1C

## Results and Discussion

The example used in this study is the monthly consumer expenditure on food in 1985, in the Netherlands, which is modeled using Model (1) and the so-called expenditure index panel conducted by Infomart, a marketing research agency [11]. We restrict analysis to *ξ*_1_ = *ξ*_2_ = … = *ξ*_12_ (annual average). The maximum likelihood estimate of *ρ* in Eq (1) for food is 0.76, with standard error 0.005 [11]. From [19], the survey cost was estimated to be roughly USD 513,000. The average cost of observing every individual in cross-section *p*_1_ and the average cost of observing every individual in panels *p*_2_ were estimated to be roughly USD 125 and USD 75, respectively. The following results are obtained using MATLAB.

### OATD method

The benchmarking parameter combinations of the algorithm designed in this paper are set as follows: the inner iteration number *B* = 2000, the initial temperature *E*_0_ = 10000, the temperature decrease rate *m* = 0.75 and the termination temperature *ε* = 0.0001. Subsequently, we analyze the effects of these parameters.

**Fig 1:**
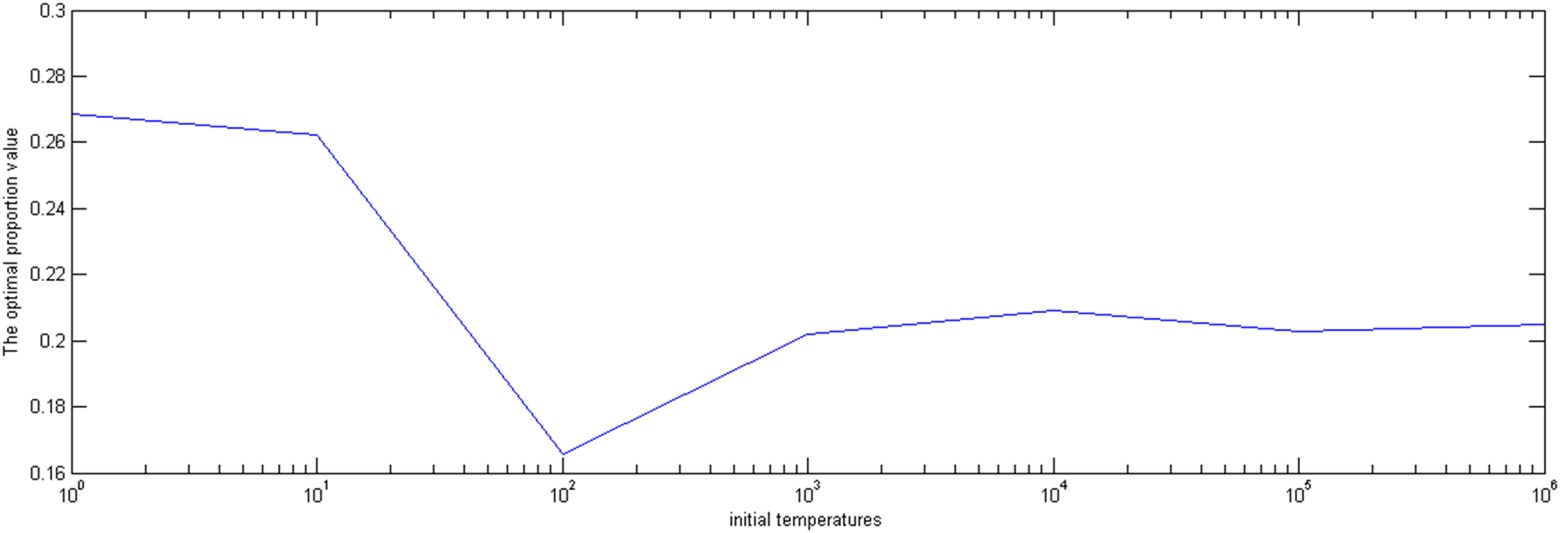
The effect of initial temperature on the optimal proportion value.

**Fig 2:**
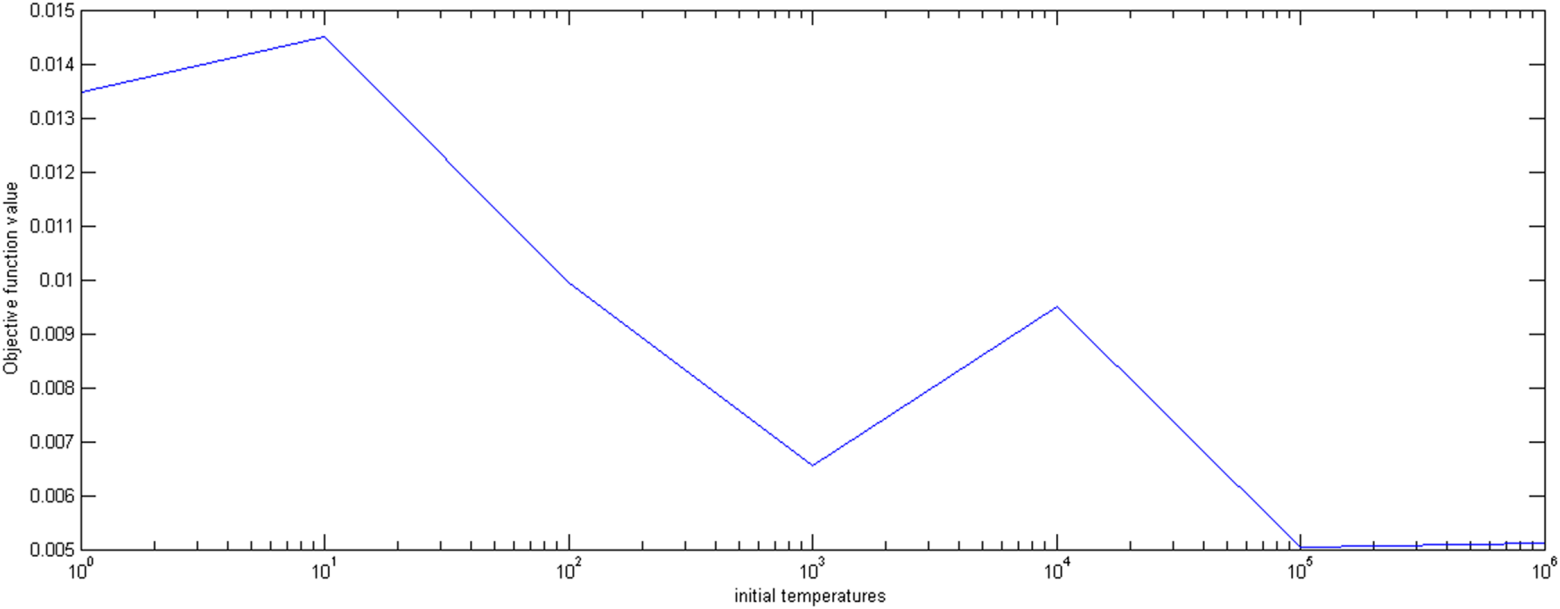
The effect of initial temperature on the objective function value.

First, we set the inner iteration number *B*, the temperature decrease rate *m*, the termination temperature *ε*, and the initial temperature *E*_0_ is changed from 1 to 1,000,000. The optimal proportion values and the corresponding objective values are shown in Fig 1. From Fig 2, the objective values fluctuate between 0.005 and 0.015 and, when the initial temperature is more than 100,000, the optimal proportion values and the corresponding objective values can converge to the optimal values of 0.2 and 0.005, respectively. Therefore, the initial temperature can be chosen between 100,000 and 1,000,000.

**Fig 3:**
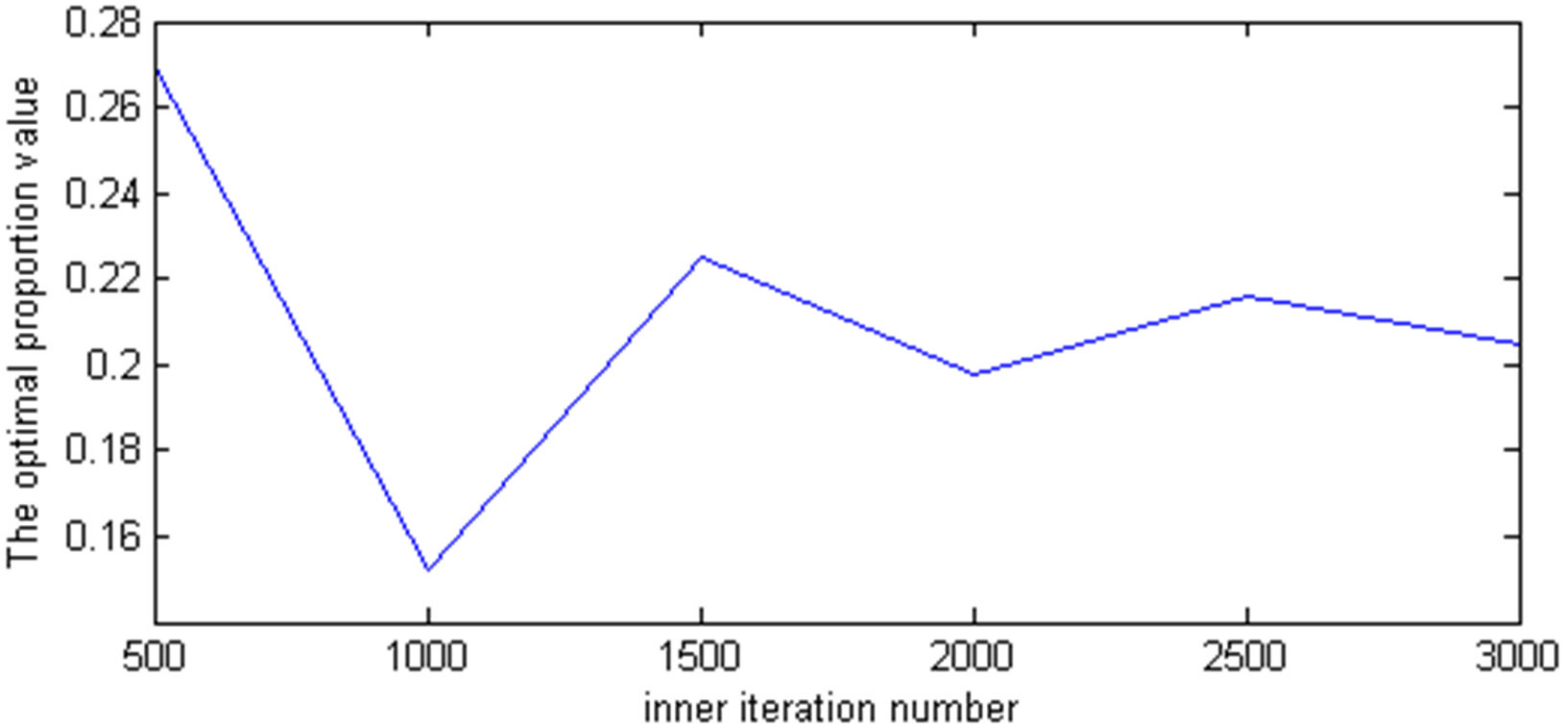
The effect of inner iteration number on the optimal proportion value.

**Fig 4:**
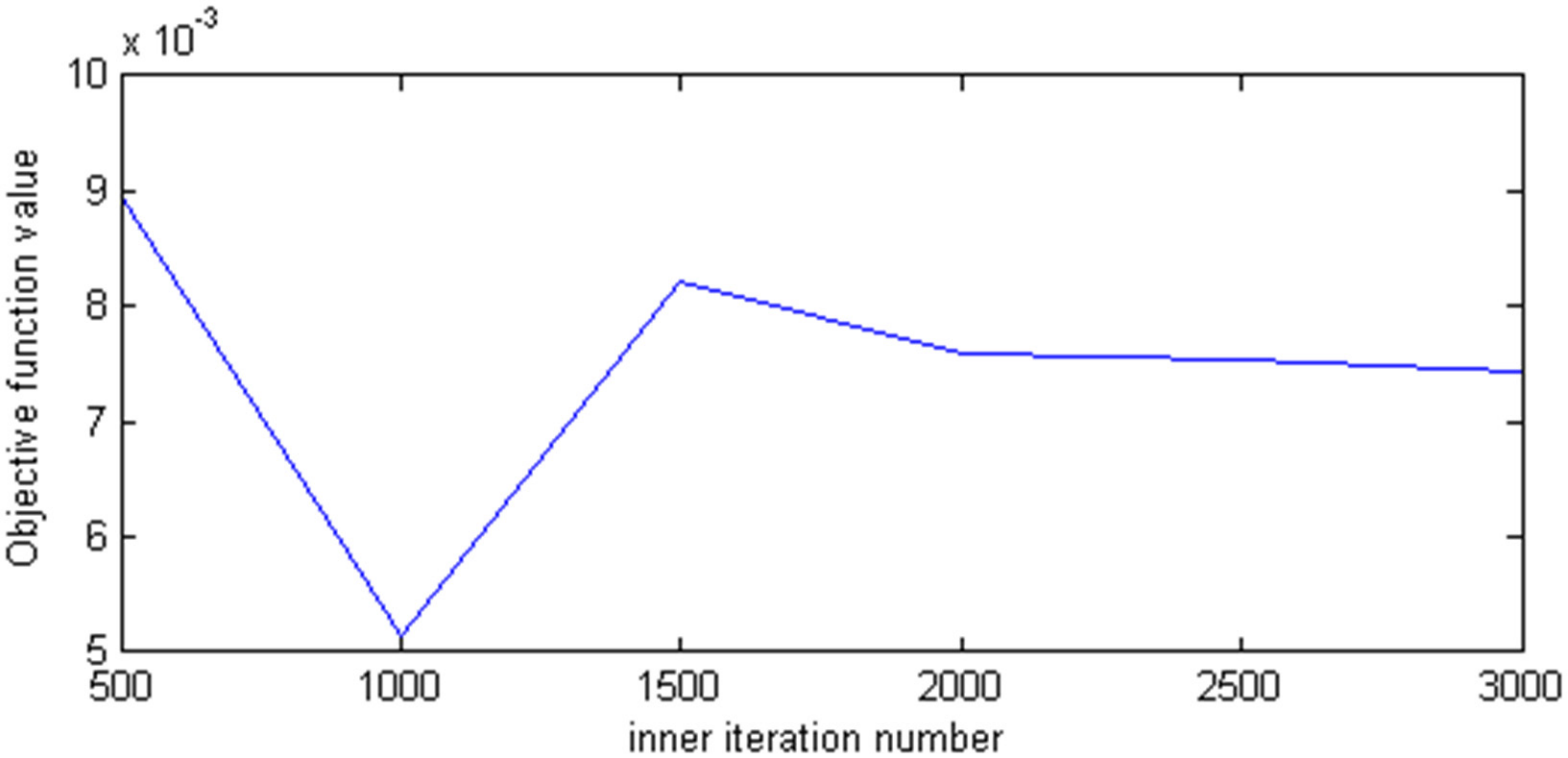
The effect of inner iteration number on the objective function value.

Second, we set the initial temperature *E*_0_, the temperature decrease rate *m*, the termination temperature *ε*, and the inner iteration number $B\frac{-b\pm \sqrt{b^{2}-4ac}}{2a}$ are changed from 500 to 3,000 to ensure that the algorithm reaches the balanced state. The optimal proportion values and the corresponding objective values are shown in Fig 3. From Fig 3, the higher the inner iteration number is, the more easily the algorithm moves from the local optimal value and converges to the global optimal value. Conversely, the higher the inner iteration number is, the longer the implementation time. In this study, the implementation time based on the set of parameters is in 10 minutes. As such, we do not consider that the inner iteration numbers increases implementation time. From Fig 4, when the inner iteration number is more than 2,000, the objective values and the optimal proportion values converge to 0.007 and 0.2, respectively. Therefore, the inner iteration number can be chosen between 2,000 and 2,500.

**Fig 5:**
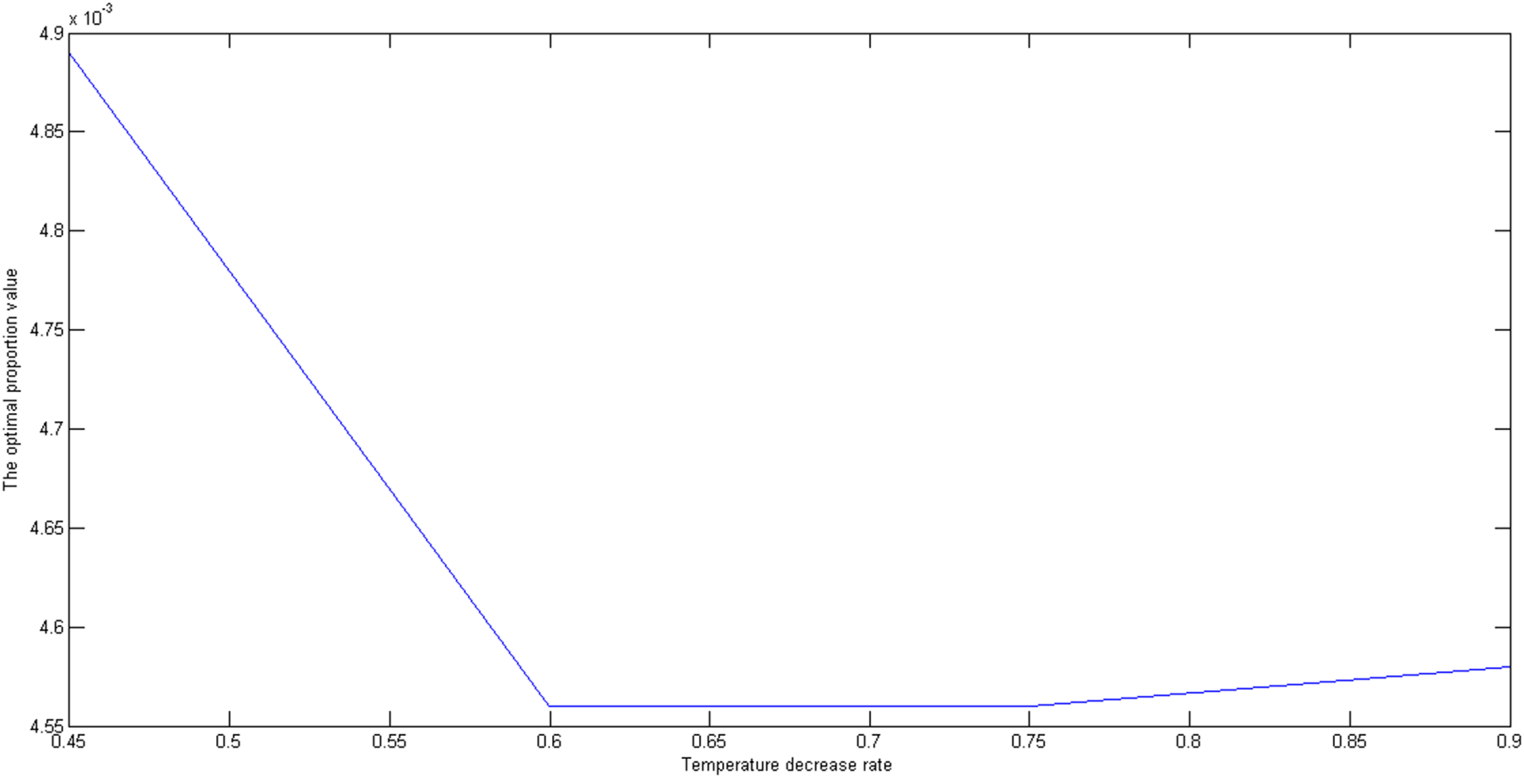
The effect of temperature decrease on the optimal proportion value.

**Fig 6:**
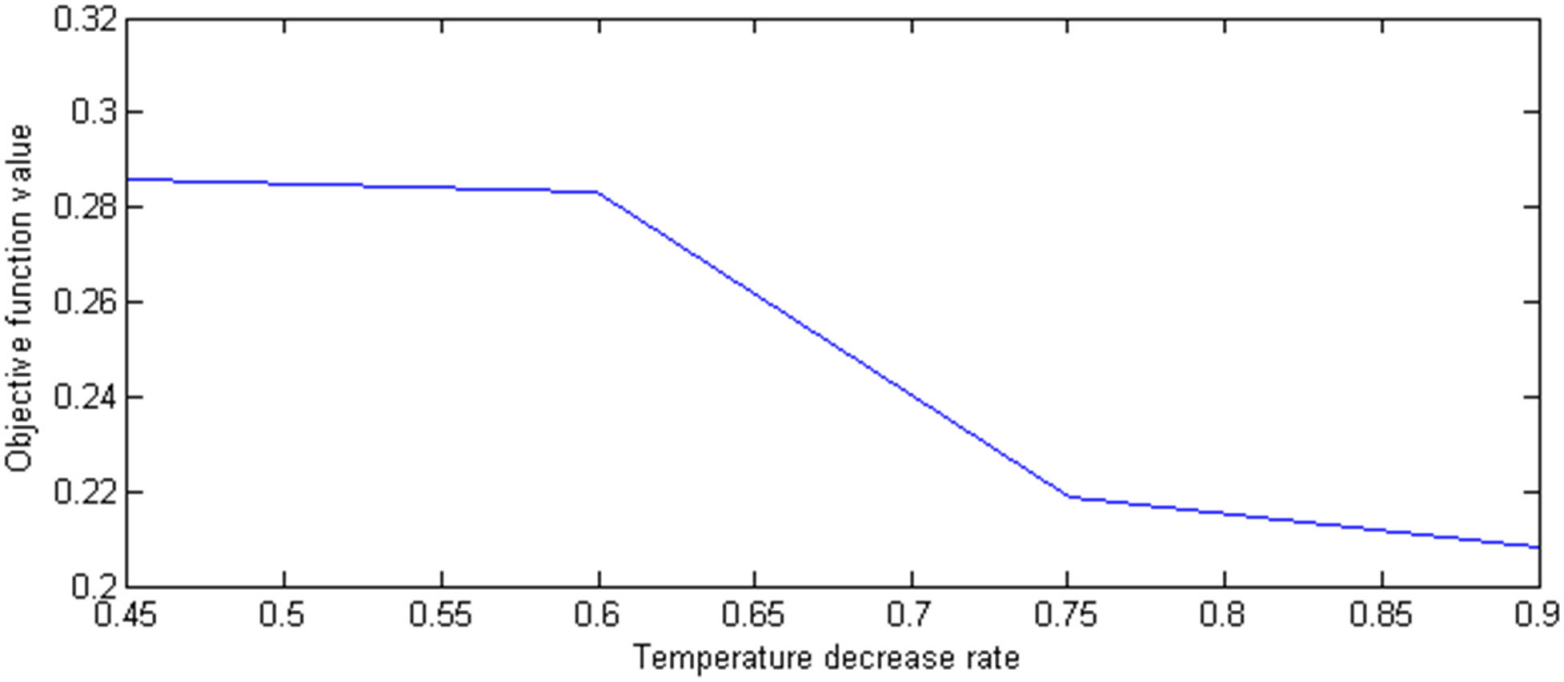
The effect of temperature decrease on the objective function value.

Subsequently, we set the initial temperature *E*_0_, the termination temperature *ε*, the inner iteration number *B*, and the temperature decrease rate *m* is changed from 0.45 to 0.9. The optimal proportion values and the corresponding objective values are shown in Fig 5. If the temperature decrease rate is less than 0.6, the algorithm falls into the local optimal value. When the temperature decrease rate is more than 0.6, the algorithm is above the local optimal value and converges to the global optimal value. As is shown in Figs 5 and 6, the optimal proportion values and the corresponding objective values converge to 0.2 and 0.0046, respectively. The temperature decrease rate determines the searching space and the larger the temperature decrease rate is, the greater the searching space. Consequently, we can choose the temperature decrease rate between 0.75 and 0.9.

**Fig 7:**
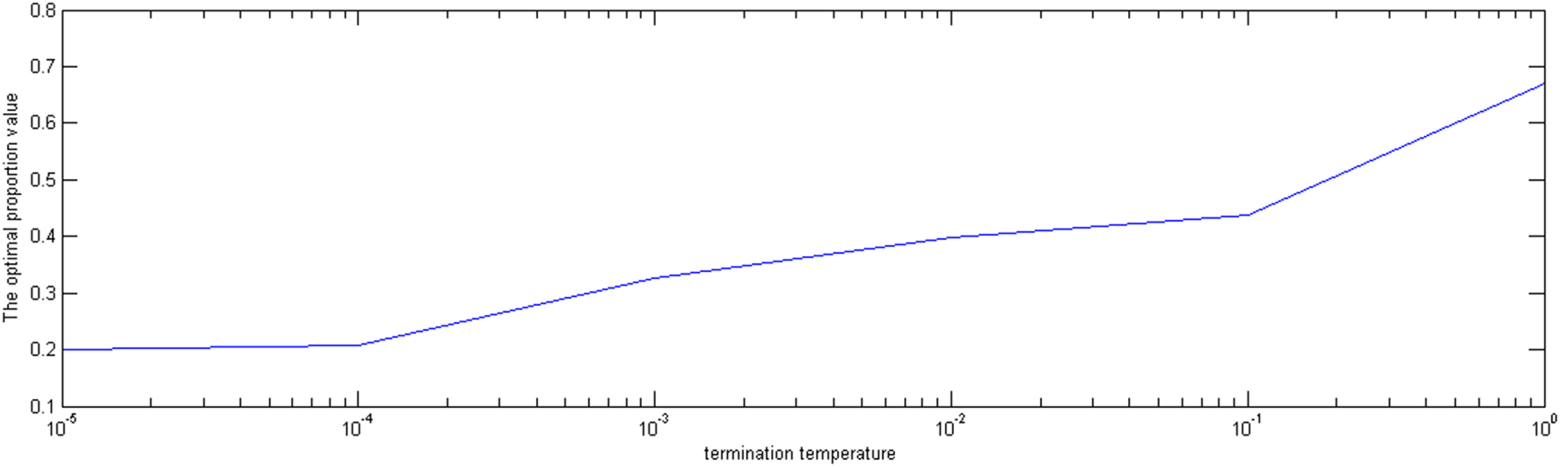
The effect of termination temperature on the optimal proportion value.

**Fig 8:**
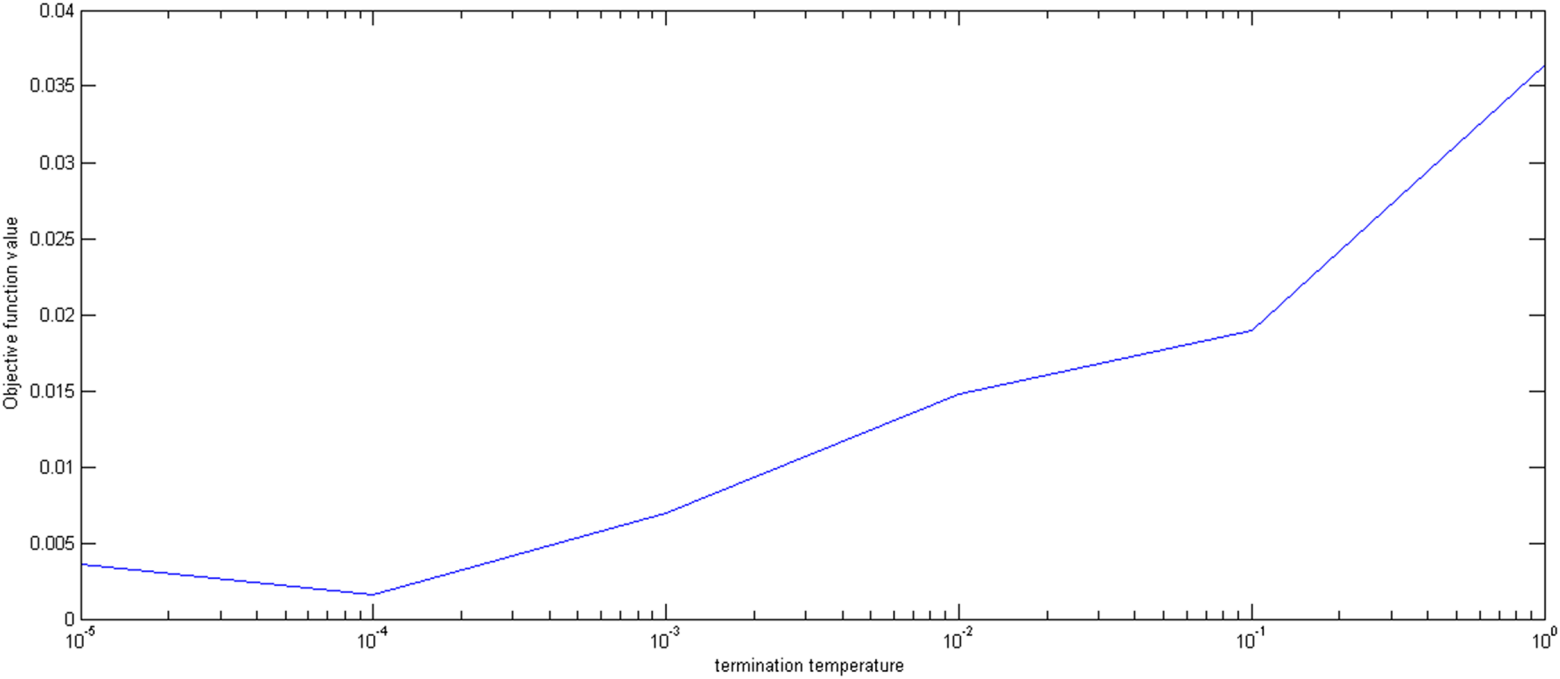
The effect of termination temperature on the objective function value.

Finally, we set the initial temperature *E*_0_, the temperature decrease rate *m*, the inner iteration number *B*, and the termination temperature *ε* is changed from 0.00001 to 1. Figs 7 and 8 show that when the termination temperature *ε* is near 0.0001, the optimal proportion values and the corresponding objective values converge to 0.2 and 0.0043, respectively. The lower the termination temperature is, the more adequate the time to converge to the optimal value. Therefore, the termination temperature can be chosen between 0.0001 and 0.00001.

### FD method

According to the above discussion, we separate the algorithm designed in this paper into two cases to analyze, the best and worst case. Under the two different cases, the fields of parameters based on the above OATD analysis can be separated into two levels (+ and -) as shown in Table 2.

**Table 2:** Parameters levels.

*Case Parameter level*		*B*	*ε*	*m*
***The best***	***+***	***[100000*,*1000000]***	***[0*.*00001*,*0*.*0001]***	***[0*.*85*,*0*.*90]***
	***-***	***[10000*,*100000]***	***[0*.*0001*,*0*.*001]***	***[0*.*80*,*0*.*85]***
***The worst***	***+***	***[1000*,*10000]***	***[0*.*001*,*0*.*1]***	***[0*.*75*,*0*.*80]***
	***-***	***[100*,*1000]***	***[0*.*01*,*0*.*1]***	***[0*.*60*,*0*.*75]***

For each case, the temperature decrease rate *m*, the inner iteration number *B* and the termination temperature *ε* are chosen randomly from their two parameter levels, and there are six values in all. Under each parameter combination, we take the optimal value 10 times the average value. The initial temperature is considered constant as compared to the whole field, since the local optimal value because of the initial temperature is very small, and the resultant errors from the initial temperature are smaller than that from other parameters. Comparing to other parameters, the initial temperature has a low quantitative influence on the algorithm designed in this paper, and will not be analyzed as such.

It is found as per Table 3 that the effects of the temperature decrease rate *m* and the inner iteration number *B* are clear, meaning that the choice of the two parameters decides whether the algorithm can obtain the optimal value. In different cases, the temperature decrease rate and the inner iteration number have different effects on the optimal value. In the best case, the inner iteration number is large enough to guarantee the solutions are stable and, if it continues to increase, the solutions do not improve much. In this case, the adjustment of temperature decrease rate can continue to narrow the neighborhood range for more convergent results. Therefore, the temperature decrease rate influences the best case. In the worst case, the inner iteration number has a significant impact, since the lower inner iteration number causes a wider searching range, which makes the process of searching far from the optimal solution. This time, the result is easier to move to the local optimal solution with the adjustment of the temperature decrease. Furthermore, Table 3 shows that the combination effect of the temperature decrease rate and the inner iteration number is also clear.

**Table 3:** Index effects on numerical results.

Parameters level							The best	The worse
Run	*B*	*m*	*ε*	(*B*,*ε*)	*(B*,*m)*	*(m*,*ε)*	y	y
**1**	**-**	**-**	**-**	**+**	**+**	**+**	**0.2100**	**0.2624**
**2**	**+**	**-**	**-**	**-**	**-**	**+**	**0.1958**	**0.2518**
**3**	**-**	**+**	**-**	**-**	**+**	**-**	**0.2020**	**0.2280**
**4**	**+**	**+**	**-**	**+**	**-**	**-**	**0.2190**	**0.2308**
**5**	**-**	**-**	**+**	**+**	**-**	**-**	**0.2030**	**0.2468**
**6**	**+**	**-**	**+**	**-**	**+**	**-**	**0.1993**	**0.2217**
**7**	**-**	**+**	**+**	**-**	**-**	**+**	**0.2315**	**0.2970**
**8**	**+**	**+**	**+**	**+**	**+**	**+**	**0.2301**	**0.2860**
	**-0.0260**	**-0.0393**	**-0.0120**	**0.0221**	**-0.0538**	**0.0112**	**The best**	**Index**
	**-0.0310**	**-0.0460**	**-0.0078**	**0.0230**	**-0.0760**	**0.0218**	**The worse**	

## Conclusions

In this paper, we discuss how to determine the optimal proportion of a series of cross-sections in all samples to minimize survey design efficiency in the analysis-of-variance model, which can be applied directly in sampling. First, we derive a theorem for choosing the optimal proportion of a series of cross-sections in all samples, irrespective of the parameters of interest and budget. In addition, our results show that, compared to a pure series of cross-sections or pure panel, the gains from choosing split panel can be substantial. Second, the efficiency of split panel design given a budget is considered and an efficient algorithm is designed to solve the constrained nonlinear integer optimization associated with the efficiency of survey designs on a budget. We further apply OATD and FD methods to analyze and compare the quantitative influence of different selections of parameters in the implementation of the algorithm with an empirical example concerning monthly consumer expenditure on food in 1985, in the Netherlands, and obtain the efficient ranges of the algorithm parameters to ensure a good solution.

For further research, we will extend the results to a more general analysis of the covariance model and derive the expressions for the variances of efficient parameter estimators. At the same time, other algorithms can be to solve the new nonlinear programming from the optimal split design in the analysis of the covariance model.

## Appendix 1

### Appendix 1A. Proof of constructing the orthogonal matrix *Q*

Proof:

$$\begin{array}{l} Q^{T}Q={\left[\frac{1}{\left(\varsigma ,\varsigma )-(\varsigma ,\beta \right)}\right]}^{2}\{(\varsigma -\beta ){\left(\varsigma -\beta \right)}^{T}(\varsigma -\beta ){\left(\varsigma -\beta \right)}^{T}\\ -2\left[\left(\varsigma ,\varsigma )-(\varsigma ,\beta \right)\right](\varsigma -\beta ){\left(\varsigma -\beta \right)}^{T}+{\left[\left(\varsigma ,\varsigma )-(\varsigma ,\beta \right)\right]}^{2}\}=I \end{array}$$ (51)

Therefore, matrix *Q* is an orthogonal matrix.

$\frac{1}{1-\rho }$ is the n-repeated eigenvalue of real symmetric matrix $V_{p_{{}_{0}}}^{-1}$, so

$$rank(\frac{1}{1-\rho }I-V_{p_{0}}^{-1})=1.$$ (52)

Let *ς*^*T*^ denote the nonzero row vector of $\frac{1}{1-\rho }I-V_{p_{0}}^{-1}$, so $(\frac{1}{1-\rho }I-V_{p_{0}}^{-1})X=0$ and *ς*^*T*^*X* = 0 have the same solutions. Let *B*_1_ denote the matrix removing the first column of matrix *Q*,

$$\varsigma^{T}=(a_{1},a_{2},\cdots ,a_{n})$$ (53)

and

$$\varsigma_{1}{}^{T}=(a_{2},a_{3},\cdots ,a_{n}).$$ (54)

Subsequently,

$$B_{1}=\frac{1}{\left(\varsigma ,\varsigma )-(\varsigma ,\beta \right)}\{(\varsigma -\beta )\varsigma_{1}^{T}-\left[\left(\varsigma ,\varsigma )-(\varsigma ,\beta \right)\right]\varsigma^{T}\left[\begin{array}{cccc} 0 & 0 & \cdots & 0\\ 1 & 0 & \cdots & 0\\ \vdots & & & \vdots \\ 0 & & & 1 \end{array}\right],$$ (55)

$$\begin{array}{l} \varsigma^{T}B_{1}=\frac{1}{\left(\varsigma ,\varsigma )-(\varsigma ,\beta \right)}\{\varsigma^{T}(\varsigma -\beta )\varsigma_{1}^{T}-\left[\left(\varsigma ,\varsigma )-(\varsigma ,\beta \right)\right]\varsigma^{T}\left[\begin{array}{cccc} 0 & 0 & \cdots & 0\\ 1 & 0 & \cdots & 0\\ \vdots & & & \vdots \\ 0 & & & 1 \end{array}\right]\\ =\frac{1}{\left(\varsigma ,\varsigma )-(\varsigma ,\beta \right)}\{\varsigma^{T}(\varsigma -\beta )\varsigma_{1}^{T}-\left[\left(\varsigma ,\varsigma )-(\varsigma ,\beta \right)\right]\varsigma_{1}^{T}\}\\ =\frac{1}{\left(\varsigma ,\varsigma )-(\varsigma ,\beta \right)}\{\varsigma^{T}(\varsigma -\beta )\varsigma_{1}^{T}-\varsigma^{T}(\varsigma -\beta )\varsigma_{1}^{T}\}=0_{1\times n-1} \end{array}.$$ (56)

As such, the bottom *n*−1 column vectors of matrix *Q* are the solutions of *ς*^*T*^*X* = 0. Matrix *Q* is an orthogonal matrix and it is impossible for all the column vectors of matrix *Q* to be zero, therefore, the bottom *n*−1 column vectors of matrix *Q* are eigenvectors belonging to eigenvalue $\frac{1}{1-\rho }$ of matrix $V_{p_{0}}^{-1}$. The *n* column vectors of the orthogonal matrix constitute a unit orthogonal vector group, and the bottom *n*−1 column vectors are unit orthogonal vector group constituted of *n*−1 eigenvectors belonging to eigenvalue $\frac{1}{1-\rho }$ of matrix $V_{p_{0}}^{-1}$. The first column vector of matrix *Q* is a unit eigenvector belonging to eigenvalue $\frac{1}{1+(T-1)\rho }$ of matrix $V_{p_{0}}^{-1}$. Moreover, different eigenvectors belonging to different eigenvalues are orthogonal, and the unit vector that is orthogonal with the bottom *n*−1 column vectors of matrix *Q* is unit eigenvector belonging to eigenvalue $\frac{1}{1+(T-1)\rho }$ of matrix $V_{p_{0}}^{-1}$. Additionally, matrix *Q* is an orthogonal matrix, and the first column vector of matrix *Q* is the unit eigenvector belonging to eigenvalue $\frac{1}{1+(T-1)\rho }$ of matrix $V_{p_{0}}^{-1}$.

### Appendix 1B. Proof of theorem 1

Proof:

Let

$$M(\lambda )=\frac{1+(T-1)\rho }{1+(T-1)\lambda \rho }\delta_{1}^{2}+\frac{1-\rho }{1-\lambda \rho }\sum_{t=2}^{T}\delta_{t}^{2},$$ (57)

$$A=\left[1+(T-1)\rho \right]\delta_{1}^{2},$$ (58)

and

$$B=(1-\rho )\sum_{t=2}^{T}\delta_{t}^{2}.$$ (59)

Subsequently,

$$M(\lambda )=\frac{A}{1+(T-1)\lambda \rho }+\frac{B}{1-\lambda \rho }.$$ (60)

Therefore,

$${M^{\prime }}_{\lambda }(\lambda )=\frac{(1-T)\rho A{\left(1-\lambda \rho \right)}^{2}+B\rho {\left(1+(T-1)\lambda \rho \right)}^{2}}{{\left(1+(T-1)\lambda \rho \right)}^{2}{\left(1-\lambda \rho \right)}^{2}}.$$ (61)

Let

$$k(\lambda )=(1-T)\rho A{\left(1-\lambda \rho \right)}^{2}+B\rho {\left(1+(T-1)\lambda \rho \right)}^{2},$$ (62)

thus

$${k^{\prime }}_{\lambda }(\lambda )=2\rho^{2}(T-1)(1-\lambda \rho )A+2B\rho^{2}(T-1)(1+(T-1)\lambda \rho ),$$ (63)

when *λ*∈[0,1],${k^{\prime }}_{\lambda }\geq 0$.

In detail,

k(λ)=((1−T)ρ3A+B(1−T)ρ23)λ2+(2Bρ2(T−1)−2(1−T)ρ2A)λ+(1−T)ρA+Bρ. (64)

Let

$$a=(1-T)\rho^{3}A+B{\left(1-T\right)}^{2}\rho^{3},$$ (65)

$$b=2B\rho^{2}(T-1)-2(1-T)\rho^{2}A,$$ (66)

c=(1−T)ρA+Bρ. (67)

Subsequently,

$$k(\lambda )=a\lambda^{2}+b\lambda +c.$$ (68)

Let Δ_*k*_ = *b*^2^−4*ac*, and as a result

Δk=4(1−ρ)∑t=2Tδt2(1+(T−1)ρ)δ12ρ4(T−1)T≥20,(λ∈[0,1]). (69)

From the above results,

When *λ*∈[0,1], *c*<0 and *k*(1)<0, M(1) is the minimum value of M(*λ*).

When *λ*∈[0,1] and *c*>0, *M*(0) is the minimum value of M(*λ*).

When *λ*∈[0,1], *a*<0, *c*<0, and *k*(1)>0, *M*(*k*_*r*_) is the minimum value of *M*(*λ*), where *k*_*r*_ is the right root of *k*(*λ*).

When *λ*∈[0,1], *a*>0, *c*<0, and *k*(1)>0, *M*(*k*_*l*_) is the minimum value of *M*(*λ*), where *k*_*l*_ is the left root of *k*(*λ*).

When *a* = 0, and then *c*<0 and *b*<0, $\text{min}\left\{M(-\frac{c}{b}),M(1)\right\}$ is the minimum value of *M*(*λ*).

### Appendix 1C. Detailed design process of proposed algorithm

Simulated annealing begins with an initial solution, and then randomly generates a neighboring solution or by using a pre-specified rule. It is the process when a state moves from the initial solution to a candidate solution in which the energy is minimized based on the Metropolis acceptance criterion. As such, we can accept the candidate solution based on the acceptance probability. We consider a number of conditions, and, subsequently, the steps of the proposed algorithm are shown.

In order to converge to the global optimal solution, we choose

$$\eta \leq P_{a}(Y^{K}|X^{K},T_{K})$$ (70)

as the acceptance criterion, which is called Metropolis criterion, where *n* is a random number distributed uniformly over [0,1] and the state acceptance function is denoted by

$$P_{a}(Y^{K}|X^{K},T_{K})=\text{min}\left\{1,\text{exp}(\frac{f(X^{K})-f(Y^{K})}{\beta T_{K}})\right\};$$ (71)

*f* is the objective function of (P2); *X*^*K*^ and *Y*^*K*^ are the current iteration point and the new iteration point, respectively; *T*_*K*_ is the k time algorithm stage temperature, which is obtained from the cooling schedule presented in Eq (50); *β* is a positive constant. In order to guarantee the integer optimal solution, the new iteration point *Y*^*K*^ is generated by the following process:

$$Y^{K}=X^{K}+\langle {\left(\frac{-1}{2}\right)}^{l}\cdot Z^{K}\rangle ,l=1,2,\cdots ,N_{1},$$ (72)

where

$$z_{i}^{K}=\langle sign(U_{i})\cdot T_{K}\cdot (\frac{1}{{\left|U_{i}\right|}^{m}}-1)\rangle ,i=1,2,\cdots ,n,$$ (73)

and $z_{i}^{K}$ is the ith component of the random vector *Z*^*K*^; *U*_1_,*U*_2_,…,*U*_*n*_ is a group of random variables distributed uniformly over [−1,1], which are independent of each other; *sign*(⋅) is the sign function; 〈⋅〉 shows the symbol of rounding numbers.

The performance and convergence of simulated annealing are crucial, and are affected by the cooling schedule. If *T* decreases fast, a fast convergence can be obtained. However, the simulated annealing reaches the global optimal solution with difficulty if *T* decreases fast. Therefore, the following cooling schedule guarantees the global optimal solution by the above theory:

$$T_{K+1}=\frac{T_{0}}{{\left(K+1\right)}^{m}},K=0,1,2\cdots ,$$ (74)

where *T*_0_ is an initial temperature; *m*≥1 and *m* is an integer constant, which determines the speed of the decreasing temperature. The choice of *T*_0_ may be crucial, the sophisticated techniques being discussed by Van Laarhoven and Aarts [12].

In conclusion, the stopping criterion for the simulated annealing algorithm is given by

$$\left|f(X^{K+1})-f(X^{K})\right|<\varepsilon ,$$ (75)

where *ε* denotes any small number.
